# A thank you note to our reviewers

**DOI:** 10.1002/jgf2.749

**Published:** 2024-11-15

We would like to express our deepest gratitude to all the individuals who have provided their valuable time and expertise to support *Journal of General and Family Medicine*. The Editorial Board wishes to acknowledge with gratitude the following Reviewers for reviewing manuscripts during the past year.

Abe, Masanori

Aihara, Hidetoshi

Ainoda, Yusuke

Akashi, Yusaku

Akiyama, Yutaro

Al‐doud, Mohammad A.

Ando, Takayuki

Aoki, Takuya

Asakura, Kentaro

Cakiroglu, Basri

Cavanna, Luigi

Cheney, Kate

Dehghani, Mansoureh

Dohi, Eisuke

Fujikawa, Hirohisa

Fujimori, Daisuke

Fujita, Kohei

Fujiwara, Motoshi

Fukase, Ryu

Fukuchi, Takahiko

Fukumori, Norio

Funakoshi, Hiraku

Funakoshi, Tomofumi

Goudarzi, Kimia

Gupta, Tarun Sen

Hagiwara, Shotaro

Hakozaki, Michiyuki

Hamasuna, Ryoichi

Hamizan, Aneeza Wan

Harada, Taku

Harada, Yukinori

Haruta, Junji

Haseda, Maho

Hashizume, Naoki

Hasunuma, Naoko

Hayase, Naoki

He, Qiushui

Hinata, Yuki

Hirayama, Yoko

Hirose, Masahiro

Ho, Roger C

Honda, Takanori

Horibata, Ken

Hotta, Harumi

Iguchi, Seitaro

Ikeda, Takaaki

Ikemoto, Tatsunori

Inada, Haruhiko

Inoue, Kazuo

Inoue, Kazuoki

Inoue, Machiko

Inoue, Yoshie

Inui, Akihiro

Ishikane, Masahiro

Ishikawa, Yukiko

Ishimaru, Hiroyasu

Ishizuka, Kosuke

Isik, Arda

Isse, Naohi

Ito, Junko

Iwama, Itaru

Iwata, Hiroyoshi

Joshita, Satoru

Kakeya, Hiroshi

Kanakubo, Yusuke

Kaneko, Makoto

Kanke, Satoshi

Kanno, Tetsuya

Kanzawa, Yohei

Kashiwagi, Shinichiro

Kataoka, Yuki

Katayama, Kohta

Kato, Daisuke

Kato, Koki

Katsuya, Tomohiro

Kawada, Shogo

Kawamoto, Ryuichi

Kawashima, Atsushi

Kenzaka, Tsuneaki

Kera, Takeshi

Kimura, Takuma

Kizawa, Yoshiyuki

Kobayashi, Hiroyuki

Kobayashi, Ryota

Koike, Soichi

Komagamine, Junpei

Kosaka, Shintaro

Kubo, Toru

Kurashige, Takashi

Kwon, Chan‐Young

Li, Yu

Maeno, Tetsuhiro

Masumoto, Shoichi

Masuzawa, Yuko

Matsubara, Shigeki

Matsumoto, Monami

Matsuyama, Yasushi

Mayumi, Toshihiko

Mezawa, Hidetoshi

Midlöv, Patrik

Mitsuhashi, Toshiharu

Miyagami, Taiju

Miyamori, Daisuke

Miyazawa, Asako

Miyoshi, Tomoko

Mizuno, Atsushi

Mizutani, Yoshinori

Molina, Gabriel A

Morales, Mignodel M

Mori, Ryuji

Morikawa, Toru

Motomura, Kazuhisa

Mughal, Faraz

Murakami, Minoru

Nagami, Haruhiko

Nagasaki, Kazuya

Nakabayashi, Masaki

Nakae, Hajime

Nakamura, Mieko

Nakhjavani, Manouchehr

Narita, Masashi

Narumoto, Keiichiro

Nermin, Ocaktan

Nishiguchi, Sho

Nishimura, Yoshito

Nishioka, Daisuke

Nishisako, Hisashi

Nishizaki, Yuji

Noguchi‐Watanabe, Maiko

Nojima, Tsuyoshi

Oguro, Hiroaki

Ohira, Yoshiyuki

Ohta, Daisuke

Ohta, Ryuichi

Okami, Yukiko

Okazaki, Yuji

Okugawa, Shu

Ooya, Yoshitaka

Otsuka, Fumio

Otsuka, Yuki

Oura, Makoto

Ozenci, Volkan

Ozone, Sachiko

Phenwan, Tharin

Prabandari, Yayi

Rajendran, Vinoth

Rezabakhsh, Aysa

Rodrıguez‐Cuadrado, Francisco

Roumen, Rudi

Sahker, Ethan

Saijo, Masayuki

Sairenji, Tomoko

Sasaki, Sho

Sasanuma, Hirotoshi

Sato, Kotaro

Satoi, Yoshinao

Shibata, Ayako

Shigenobu, Yuya

Shimamura, Yoshinosuke

Son, Daisuke

Sugiyama, Takehiro

Suzuki, Keisuke

Suzuki, Masao

Suzuki, Risa

Suzuki, Yasuyuki

Tago, Masaki

Takahashi, Fumihiko

Takahashi, Kenzo

Takahashi, Masaki

Takahashi, Noriyuki

Takamura, Akiteru

Takayama, Shin

Takegami, Yasuhiko

Takeshima, Taro

Tamiya, Nanako

Tamura, Haruka

Tanaka, Kendo

Tanaka, Masao

Tanihara, Shinichi

Tanriverdi, Zulkif

Terada, Shuhei

Tobari, Hiroko

Toda, Hiroki

Tokinobu, Akiko

Tokuda, Koichi

Tsuchida, Tomoya

Tsugihashi, Yukio

Tsutsumi, Akizumi

Tzeng, Nian‐Sheng

Uematsu, Takuya

Ukawa, Shigekazu

Wada, Mikio

Watanabe, Takamasa

Watanabe, Yukiko

Watari, Takashi

Weissman, Charles

Xu, Li‐Jun

Yabuki, Taku

Yahata, Shinsuke

Yamada, Takayuki

Yamaguchi, Yoshiko

Yamakita, Mitsuya

Yamamoto, Koichi

Yamamoto, Shungo

Yamamoto, Yu

Yamanashi, Hirotomo

Yamashita, Shun

Yano, Takahisa

Yasumoto, Hiroaki

Yokokawa, Daiki

Yokokawa, Hirohide

Yokoya, Shoji

Yoshida, Shuhei

Yoshimoto, Kiyomi

Zaitsu, Masayoshi

Zukeran, Sota

